# 2-Ethyl-3,5,6-triphenyl­pyrazine

**DOI:** 10.1107/S1600536812039827

**Published:** 2012-09-26

**Authors:** N. Anuradha, A. Thiruvalluvar, S. Chitra, D. Devanathan, R. J. Butcher

**Affiliations:** a Postgraduate Research Department of Physics, Rajah Serfoji Government College (Autonomous), Thanjavur 613 005, Tamilnadu, India; b Department of Chemistry, KSR College of Engineering, KSR Kalvi Nagar, Tiruchengode 637 215, Tamilnadu, India; c Department of Chemistry, Government Arts College, C. Mutlur 608 102, Chidambaram, Tamilnadu, India; d Department of Chemistry, Howard University, 525 College Street NW, Washington, DC 20059, USA

## Abstract

In the title mol­ecule, C_24_H_20_N_2_, the pyrazine ring is significantly distorted from planarity, presumably due to steric crowding, and its conformation is well described as a flattened twist-boat. The benzene ring adjacent to the ethyl group forms dihedral angles of 53.79 (13) and 85.47 (12)° with the other benzene rings; the dihedral angle between adjacent benzene rings is 57.90 (12)°. The ethyl group is disordered over two positions; the site-occupancy factor of the major component is 0.546 (4). No hydrogen bonds are found in the crystal structure.

## Related literature

For the biological properties of pyrazines and for a closely related crystal structure, see: Anuradha *et al.* (2009 ▶).
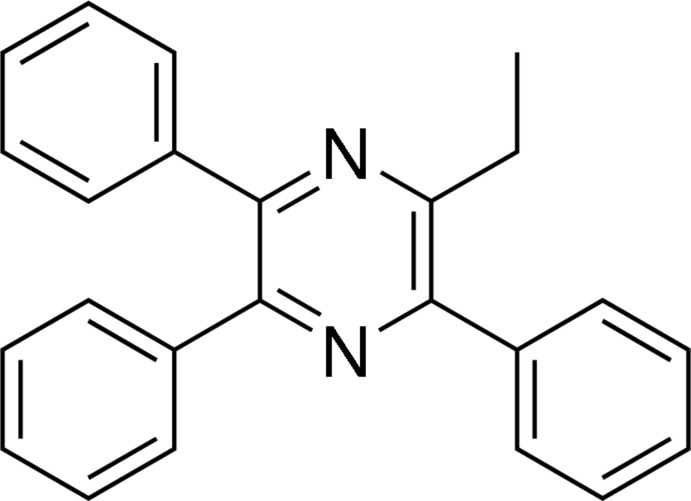


## Experimental

### 

#### Crystal data

C_24_H_20_N_2_
*M* *_r_* = 336.42
Triclinic, 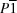

*a* = 9.2327 (9) Å
*b* = 9.8708 (11) Å
*c* = 10.6787 (14) Å
α = 79.604 (10)°
β = 70.351 (11)°
γ = 87.848 (8)°
*V* = 901.20 (19) Å^3^
*Z* = 2
Cu *K*α radiation
μ = 0.56 mm^−1^
*T* = 123 K
0.44 × 0.37 × 0.24 mm

#### Data collection

Agilent Xcalibur Ruby Gemini diffractometer
Absorption correction: multi-scan (*CrysAlis PRO*; Agilent, 2012 ▶) *T*_min_ = 0.845, *T*_max_ = 1.000
5769 measured reflections
3576 independent reflections
2622 reflections with *I* > 2σ(*I*)
*R*_int_ = 0.027

#### Refinement

*R*[*F* ^2^ > 2σ(*F* ^2^)] = 0.063
*wR*(*F* ^2^) = 0.198
*S* = 1.05
3576 reflections
244 parameters
H-atom parameters constrained
Δρ_max_ = 0.29 e Å^−3^
Δρ_min_ = −0.21 e Å^−3^

### 

Data collection: *CrysAlis PRO* (Agilent, 2012 ▶); cell refinement: *CrysAlis PRO*; data reduction: *CrysAlis PRO*; program(s) used to solve structure: *SHELXS97* (Sheldrick, 2008 ▶); program(s) used to refine structure: *SHELXL97* (Sheldrick, 2008 ▶); molecular graphics: *ORTEP-3* (Farrugia, 1997 ▶); software used to prepare material for publication: *PLATON* (Spek, 2009 ▶).

## Supplementary Material

Crystal structure: contains datablock(s) global, I. DOI: 10.1107/S1600536812039827/tk5151sup1.cif

Structure factors: contains datablock(s) I. DOI: 10.1107/S1600536812039827/tk5151Isup2.hkl

Supplementary material file. DOI: 10.1107/S1600536812039827/tk5151Isup3.cdx

Supplementary material file. DOI: 10.1107/S1600536812039827/tk5151Isup4.cml

Additional supplementary materials:  crystallographic information; 3D view; checkCIF report

## Figures and Tables

**Fig. 1.**
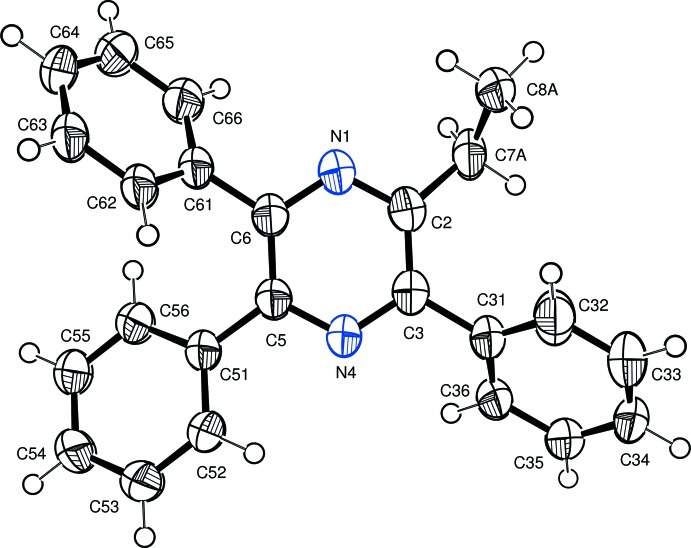
The molecular structure of the title compound, with displacement ellipsoids drawn at the 50% probability level. H atoms are shown as small spheres of arbitrary radius. Only the major disorder component of ethyl group is shown.

## supplementary crystallographic information

### Comment

As part of our investigations of pyrazine derivatives (Anuradha *et al.*, 2009) to compare their chemical and biological activities, we have undertaken the X-ray crystal structure analysis of the title compound.

In the title molecule, Fig.1, the pyrazine ring adopts a flattened twist-boat conformation. The phenyl ring at position 5 makes a dihedral angle of 53.79 (13)° and 57.90 (12)° with the phenyl rings at position 3 and 6 respectively. The dihedral angle between the phenyl rings at positions 3 and 6 is 85.47 (12)°. The ethyl group is found to be disordered over two positions; the site occupancy factors refined to 0.546 (4) and 0.454 (4). No classical hydrogen bonds are found in the crystal structure.

### Experimental

To a homogeneous solution of benzil (1.05 g, 0.005 mol) and 1-ethyl-2-phenyl-1,2-ethanediaminedihydrochloride (1.45 g, 0.005 mol) in ethanol (20 ml), sodium acetate trihydrate (2.04 g, 0.015 mol) was added. The precipitated sodium chloride was filtered off and the filtrate was refluxed for 2 h. On completion of the reaction, as indicated by TLC, the reaction mixture was poured into crushed ice and the resulting solid was filtered and purified by column chromatography on silica gel. Elution with benzene–petroleum ether (3:2 *v*/*v*) at 333–353 K gave the pure product. Yield 1.54 g (70%). The pure product was recrystallized in ethyl acetate, to obtain crystals suitable for X-ray diffraction studies.

### Refinement

The H atoms were positioned geometrically and allowed to ride on their parent atoms, with C—H = 0.95–0.99 Å , and with *U*_iso_(H) = 1.2–1.5*U*_eq_(C). The ethyl group is found to be disordered over two positions. The anisotropic displacement parameters of equivalent atoms were constrained to be equal; the site occupancy factors refined to 0.546 (4) and 0.454 (4).

### Crystal data

**Table:**

C_24_H_20_N_2_	*Z* = 2
*M**_r_* = 336.42	*F*(000) = 356
Triclinic, *P*1	*D*_x_ = 1.240 Mg m^−^^3^
Hall symbol: -P 1	Melting point: 423 K
*a* = 9.2327 (9) Å	Cu *K*α radiation, λ = 1.54184 Å
*b* = 9.8708 (11) Å	Cell parameters from 1596 reflections
*c* = 10.6787 (14) Å	θ = 4.6–76.1°
α = 79.604 (10)°	µ = 0.56 mm^−^^1^
β = 70.351 (11)°	*T* = 123 K
γ = 87.848 (8)°	Prism, colourless
*V* = 901.20 (19) Å^3^	0.44 × 0.37 × 0.24 mm

### Data collection

**Table:**

Agilent Xcalibur Ruby Gemini diffractometer	3576 independent reflections
Radiation source: Enhance (Cu) X-ray Source	2622 reflections with *I* > 2σ(*I*)
Graphite monochromator	*R*_int_ = 0.027
Detector resolution: 10.5081 pixels mm^-1^	θ_max_ = 76.3°, θ_min_ = 4.6°
ω scans	*h* = −11→11
Absorption correction: multi-scan (*CrysAlis PRO*; Agilent, 2012)	*k* = −6→12
*T*_min_ = 0.845, *T*_max_ = 1.000	*l* = −12→13
5769 measured reflections	

### Refinement

**Table:**

Refinement on *F*^2^	Primary atom site location: structure-invariant direct methods
Least-squares matrix: full	Secondary atom site location: difference Fourier map
*R*[*F*^2^ > 2σ(*F*^2^)] = 0.063	Hydrogen site location: inferred from neighbouring sites
*wR*(*F*^2^) = 0.198	H-atom parameters constrained
*S* = 1.05	*w* = 1/[σ^2^(*F*_o_^2^) + (0.1016*P*)^2^ + 0.1391*P*] where *P* = (*F*_o_^2^ + 2*F*_c_^2^)/3
3576 reflections	(Δ/σ)_max_ = 0.001
244 parameters	Δρ_max_ = 0.29 e Å^−^^3^
0 restraints	Δρ_min_ = −0.21 e Å^−^^3^

### Special details

**Table:**

Geometry. Bond distances, angles *etc*. have been calculated using the rounded fractional coordinates. All su's are estimated from the variances of the (full) variance-covariance matrix. The cell e.s.d.'s are taken into account in the estimation of distances, angles and torsion angles
Refinement. Refinement of *F*^2^ against ALL reflections. The weighted *R*-factor *wR* and goodness of fit *S* are based on *F*^2^, conventional *R*-factors *R* are based on *F*, with *F* set to zero for negative *F*^2^. The threshold expression of *F*^2^ > 2σ(*F*^2^) is used only for calculating *R*-factors(gt) *etc*. and is not relevant to the choice of reflections for refinement. *R*-factors based on *F*^2^ are statistically about twice as large as those based on *F*, and *R*-factors based on ALL data will be even larger.

### Fractional atomic coordinates and isotropic or equivalent isotropic displacement parameters (Å^2^)

**Table:**

	*x*	*y*	*z*	*U*_iso_*/*U*_eq_	Occ. (<1)
N1	0.3945 (2)	0.3594 (2)	0.4200 (2)	0.0631 (6)	
N4	0.6170 (2)	0.19621 (19)	0.48492 (19)	0.0528 (5)	
C2	0.5434 (3)	0.3820 (3)	0.3449 (3)	0.0645 (8)	
C3	0.6537 (3)	0.2913 (3)	0.3721 (2)	0.0580 (7)	
C5	0.4699 (2)	0.1805 (2)	0.5654 (2)	0.0498 (6)	
C6	0.3549 (3)	0.2558 (2)	0.5259 (2)	0.0534 (6)	
C7A	0.5713 (15)	0.5231 (13)	0.2548 (9)	0.063 (3)	0.546 (4)
C8A	0.4983 (5)	0.5205 (5)	0.1381 (5)	0.0663 (11)	0.546 (4)
C31	0.8188 (3)	0.2978 (2)	0.2824 (2)	0.0563 (7)	
C32	0.8599 (3)	0.3073 (3)	0.1431 (3)	0.0720 (9)	
C33	1.0135 (3)	0.3172 (3)	0.0618 (3)	0.0736 (9)	
C34	1.1255 (3)	0.3181 (3)	0.1184 (3)	0.0656 (8)	
C35	1.0868 (3)	0.3074 (3)	0.2567 (3)	0.0603 (7)	
C36	0.9331 (3)	0.2953 (2)	0.3381 (2)	0.0549 (7)	
C51	0.4413 (2)	0.0844 (2)	0.6962 (2)	0.0496 (6)	
C52	0.5414 (2)	−0.0232 (2)	0.7054 (2)	0.0542 (6)	
C53	0.5192 (3)	−0.1138 (3)	0.8258 (3)	0.0634 (8)	
C54	0.3977 (3)	−0.0970 (3)	0.9402 (2)	0.0661 (8)	
C55	0.2987 (3)	0.0105 (3)	0.9334 (2)	0.0614 (8)	
C56	0.3201 (3)	0.1008 (2)	0.8133 (2)	0.0555 (7)	
C61	0.1863 (2)	0.2299 (2)	0.5919 (2)	0.0518 (6)	
C62	0.1196 (3)	0.0985 (2)	0.6206 (2)	0.0534 (6)	
C63	−0.0386 (3)	0.0797 (3)	0.6767 (2)	0.0573 (7)	
C64	−0.1311 (3)	0.1920 (3)	0.7057 (2)	0.0609 (7)	
C65	−0.0657 (3)	0.3224 (3)	0.6778 (3)	0.0634 (8)	
C66	0.0919 (3)	0.3419 (3)	0.6198 (3)	0.0600 (7)	
C8B	0.4650 (6)	0.5857 (6)	0.2183 (6)	0.0663 (11)	0.454 (4)
C7B	0.5880 (19)	0.4955 (17)	0.2180 (12)	0.063 (3)	0.454 (4)
H2A	0.68302	0.54456	0.21418	0.0759*	0.546 (4)
H1A	0.52231	0.59471	0.30839	0.0759*	0.546 (4)
H5A	0.55242	0.45393	0.08165	0.0998*	0.546 (4)
H3A	0.38908	0.49358	0.17936	0.0998*	0.546 (4)
H4A	0.50889	0.61234	0.08217	0.0998*	0.546 (4)
H34	1.23057	0.32608	0.06258	0.0787*	
H35	1.16506	0.30835	0.29591	0.0724*	
H36	0.90676	0.28515	0.43334	0.0659*	
H52	0.62619	−0.03460	0.62782	0.0650*	
H53	0.58741	−0.18763	0.82996	0.0760*	
H54	0.38243	−0.15891	1.02299	0.0793*	
H55	0.21525	0.02227	1.01182	0.0737*	
H56	0.25193	0.17485	0.81016	0.0665*	
H62	0.18296	0.02151	0.60148	0.0640*	
H63	−0.08372	−0.00978	0.69533	0.0688*	
H64	−0.23956	0.17912	0.74471	0.0730*	
H65	−0.12926	0.39879	0.69861	0.0761*	
H66	0.13612	0.43203	0.59889	0.0720*	
H32	0.78209	0.30691	0.10332	0.0864*	
H33	1.04047	0.32336	−0.03311	0.0883*	
H6B	0.61707	0.45254	0.13633	0.0759*	0.454 (4)
H7B	0.67843	0.54886	0.21448	0.0759*	0.454 (4)
H8B	0.42780	0.61962	0.30408	0.0998*	0.454 (4)
H9B	0.50093	0.66372	0.14346	0.0998*	0.454 (4)
H10B	0.38122	0.53611	0.20745	0.0998*	0.454 (4)

### Atomic displacement parameters (Å^2^)

**Table:**

	*U*^11^	*U*^22^	*U*^33^	*U*^12^	*U*^13^	*U*^23^
N1	0.0495 (10)	0.0628 (12)	0.0572 (11)	0.0002 (8)	−0.0045 (9)	0.0135 (9)
N4	0.0437 (9)	0.0559 (10)	0.0440 (9)	0.0028 (7)	−0.0019 (7)	0.0032 (7)
C2	0.0504 (12)	0.0650 (14)	0.0596 (14)	−0.0040 (10)	−0.0071 (10)	0.0148 (11)
C3	0.0484 (12)	0.0617 (13)	0.0491 (12)	−0.0004 (9)	−0.0039 (9)	0.0037 (10)
C5	0.0461 (11)	0.0481 (11)	0.0434 (10)	0.0039 (8)	−0.0041 (8)	0.0000 (8)
C6	0.0469 (11)	0.0496 (11)	0.0490 (11)	0.0025 (8)	−0.0030 (9)	0.0027 (9)
C7A	0.056 (3)	0.067 (5)	0.046 (5)	−0.006 (3)	0.003 (4)	0.004 (3)
C8A	0.0526 (18)	0.061 (2)	0.062 (2)	0.0075 (15)	−0.0023 (16)	0.0137 (14)
C31	0.0470 (11)	0.0547 (12)	0.0492 (12)	−0.0005 (9)	−0.0011 (9)	0.0073 (9)
C32	0.0575 (14)	0.0932 (19)	0.0529 (14)	−0.0104 (13)	−0.0094 (11)	0.0032 (13)
C33	0.0674 (16)	0.0890 (19)	0.0448 (12)	0.0002 (13)	0.0012 (11)	−0.0007 (12)
C34	0.0462 (12)	0.0663 (14)	0.0578 (14)	0.0037 (10)	0.0065 (10)	0.0085 (11)
C35	0.0470 (12)	0.0590 (13)	0.0598 (13)	0.0027 (9)	−0.0077 (10)	0.0075 (10)
C36	0.0523 (12)	0.0479 (11)	0.0483 (11)	0.0042 (9)	−0.0031 (9)	0.0049 (9)
C51	0.0447 (10)	0.0514 (11)	0.0409 (10)	0.0042 (8)	−0.0038 (8)	0.0006 (8)
C52	0.0448 (11)	0.0610 (12)	0.0435 (10)	0.0097 (9)	−0.0023 (8)	−0.0018 (9)
C53	0.0590 (13)	0.0684 (14)	0.0510 (12)	0.0170 (11)	−0.0109 (10)	0.0013 (10)
C54	0.0655 (14)	0.0749 (16)	0.0421 (11)	0.0110 (12)	−0.0083 (10)	0.0089 (10)
C55	0.0559 (13)	0.0727 (15)	0.0400 (11)	0.0087 (11)	−0.0007 (9)	−0.0024 (10)
C56	0.0508 (11)	0.0591 (12)	0.0440 (11)	0.0109 (9)	−0.0035 (9)	−0.0038 (9)
C61	0.0449 (11)	0.0534 (11)	0.0410 (10)	0.0047 (8)	−0.0008 (8)	0.0047 (8)
C62	0.0487 (11)	0.0532 (12)	0.0409 (10)	0.0065 (9)	0.0008 (8)	0.0035 (8)
C63	0.0511 (12)	0.0597 (12)	0.0437 (11)	−0.0021 (9)	0.0005 (9)	0.0036 (9)
C64	0.0425 (11)	0.0780 (15)	0.0441 (11)	0.0050 (10)	0.0029 (9)	0.0001 (10)
C65	0.0527 (13)	0.0662 (14)	0.0562 (13)	0.0146 (10)	−0.0022 (10)	−0.0068 (11)
C66	0.0538 (12)	0.0553 (12)	0.0579 (13)	0.0056 (9)	−0.0062 (10)	−0.0022 (10)
C8B	0.0526 (18)	0.061 (2)	0.062 (2)	0.0075 (15)	−0.0023 (16)	0.0137 (14)
C7B	0.056 (3)	0.067 (5)	0.046 (5)	−0.006 (3)	0.003 (4)	0.004 (3)

### Geometric parameters (Å, º)

**Table:**

N1—C2	1.342 (4)	C63—C64	1.389 (4)
N1—C6	1.338 (3)	C64—C65	1.382 (4)
N4—C3	1.337 (3)	C65—C66	1.382 (4)
N4—C5	1.338 (3)	C7A—H1A	0.9900
C2—C3	1.400 (4)	C7A—H2A	0.9900
C2—C7A	1.519 (12)	C7B—H6B	0.9900
C2—C7B	1.540 (14)	C7B—H7B	0.9900
C3—C31	1.500 (4)	C8A—H5A	0.9800
C5—C6	1.409 (3)	C8A—H3A	0.9800
C5—C51	1.488 (3)	C8A—H4A	0.9800
C6—C61	1.487 (3)	C8B—H8B	0.9800
C7A—C8A	1.608 (13)	C8B—H9B	0.9800
C7B—C8B	1.416 (19)	C8B—H10B	0.9800
C31—C36	1.372 (4)	C32—H32	0.9500
C31—C32	1.392 (4)	C33—H33	0.9500
C32—C33	1.388 (4)	C34—H34	0.9500
C33—C34	1.363 (4)	C35—H35	0.9500
C34—C35	1.383 (4)	C36—H36	0.9500
C35—C36	1.389 (4)	C52—H52	0.9500
C51—C52	1.393 (3)	C53—H53	0.9500
C51—C56	1.400 (3)	C54—H54	0.9500
C52—C53	1.384 (4)	C55—H55	0.9500
C53—C54	1.384 (4)	C56—H56	0.9500
C54—C55	1.382 (4)	C62—H62	0.9500
C55—C56	1.382 (3)	C63—H63	0.9500
C61—C66	1.394 (4)	C64—H64	0.9500
C61—C62	1.395 (3)	C65—H65	0.9500
C62—C63	1.385 (4)	C66—H66	0.9500
			
C2—N1—C6	119.3 (2)	C8B—C7B—H6B	109.00
C3—N4—C5	118.9 (2)	C8B—C7B—H7B	109.00
N1—C2—C3	119.7 (3)	H6B—C7B—H7B	108.00
N1—C2—C7A	111.8 (6)	C2—C7B—H7B	109.00
N1—C2—C7B	119.3 (7)	C2—C7B—H6B	109.00
C3—C2—C7A	127.5 (6)	H3A—C8A—H5A	109.00
C3—C2—C7B	120.5 (7)	H3A—C8A—H4A	109.00
N4—C3—C2	120.7 (2)	C7A—C8A—H3A	109.00
N4—C3—C31	116.1 (2)	C7A—C8A—H4A	109.00
C2—C3—C31	123.1 (2)	C7A—C8A—H5A	109.00
N4—C5—C6	120.09 (19)	H4A—C8A—H5A	109.00
N4—C5—C51	115.46 (18)	C7B—C8B—H8B	109.00
C6—C5—C51	124.42 (18)	C7B—C8B—H10B	109.00
N1—C6—C5	119.9 (2)	H8B—C8B—H9B	109.00
N1—C6—C61	114.7 (2)	C7B—C8B—H9B	109.00
C5—C6—C61	125.43 (18)	H9B—C8B—H10B	109.00
C2—C7A—C8A	108.0 (8)	H8B—C8B—H10B	110.00
C2—C7B—C8B	111.3 (10)	C33—C32—H32	120.00
C3—C31—C32	121.7 (2)	C31—C32—H32	120.00
C3—C31—C36	119.54 (19)	C32—C33—H33	120.00
C32—C31—C36	118.7 (2)	C34—C33—H33	120.00
C31—C32—C33	120.6 (3)	C35—C34—H34	120.00
C32—C33—C34	119.9 (3)	C33—C34—H34	120.00
C33—C34—C35	120.3 (3)	C36—C35—H35	120.00
C34—C35—C36	119.8 (3)	C34—C35—H35	120.00
C31—C36—C35	120.7 (2)	C31—C36—H36	120.00
C5—C51—C52	119.45 (18)	C35—C36—H36	120.00
C52—C51—C56	118.07 (19)	C53—C52—H52	119.00
C5—C51—C56	122.43 (18)	C51—C52—H52	119.00
C51—C52—C53	121.0 (2)	C54—C53—H53	120.00
C52—C53—C54	120.1 (3)	C52—C53—H53	120.00
C53—C54—C55	119.6 (2)	C53—C54—H54	120.00
C54—C55—C56	120.5 (2)	C55—C54—H54	120.00
C51—C56—C55	120.7 (2)	C56—C55—H55	120.00
C6—C61—C62	122.11 (19)	C54—C55—H55	120.00
C62—C61—C66	119.2 (2)	C51—C56—H56	120.00
C6—C61—C66	118.6 (2)	C55—C56—H56	120.00
C61—C62—C63	120.3 (2)	C61—C62—H62	120.00
C62—C63—C64	119.9 (3)	C63—C62—H62	120.00
C63—C64—C65	120.1 (3)	C62—C63—H63	120.00
C64—C65—C66	120.2 (3)	C64—C63—H63	120.00
C61—C66—C65	120.3 (3)	C65—C64—H64	120.00
C2—C7A—H1A	110.00	C63—C64—H64	120.00
C2—C7A—H2A	110.00	C66—C65—H65	120.00
C8A—C7A—H1A	110.00	C64—C65—H65	120.00
C8A—C7A—H2A	110.00	C61—C66—H66	120.00
H1A—C7A—H2A	108.00	C65—C66—H66	120.00
			
C6—N1—C2—C3	4.6 (4)	N1—C6—C61—C66	−46.4 (3)
C6—N1—C2—C7A	−164.7 (5)	C5—C6—C61—C62	−48.8 (3)
C2—N1—C6—C5	6.2 (3)	C5—C6—C61—C66	134.3 (2)
C2—N1—C6—C61	−173.1 (2)	C3—C31—C32—C33	178.1 (3)
C5—N4—C3—C2	6.1 (4)	C36—C31—C32—C33	−1.4 (4)
C5—N4—C3—C31	−176.5 (2)	C3—C31—C36—C35	−177.1 (2)
C3—N4—C5—C6	4.8 (3)	C32—C31—C36—C35	2.5 (3)
C3—N4—C5—C51	−173.2 (2)	C31—C32—C33—C34	−0.2 (4)
N1—C2—C3—N4	−11.2 (4)	C32—C33—C34—C35	0.8 (5)
N1—C2—C3—C31	171.6 (2)	C33—C34—C35—C36	0.2 (4)
C7A—C2—C3—N4	156.3 (6)	C34—C35—C36—C31	−1.9 (4)
C7A—C2—C3—C31	−20.9 (7)	C5—C51—C52—C53	179.4 (2)
N1—C2—C7A—C8A	−70.8 (7)	C56—C51—C52—C53	1.9 (3)
C3—C2—C7A—C8A	120.9 (7)	C5—C51—C56—C55	−179.0 (2)
N4—C3—C31—C32	134.5 (2)	C52—C51—C56—C55	−1.6 (3)
N4—C3—C31—C36	−45.9 (3)	C51—C52—C53—C54	−1.2 (4)
C2—C3—C31—C32	−48.2 (4)	C52—C53—C54—C55	0.1 (4)
C2—C3—C31—C36	131.4 (3)	C53—C54—C55—C56	0.2 (4)
N4—C5—C6—N1	−11.3 (3)	C54—C55—C56—C51	0.6 (4)
N4—C5—C6—C61	167.98 (19)	C6—C61—C62—C63	−177.1 (2)
C51—C5—C6—N1	166.6 (2)	C66—C61—C62—C63	−0.3 (3)
C51—C5—C6—C61	−14.1 (3)	C6—C61—C66—C65	178.3 (2)
N4—C5—C51—C52	−27.8 (3)	C62—C61—C66—C65	1.3 (4)
N4—C5—C51—C56	149.6 (2)	C61—C62—C63—C64	−0.6 (3)
C6—C5—C51—C52	154.2 (2)	C62—C63—C64—C65	0.5 (3)
C6—C5—C51—C56	−28.4 (3)	C63—C64—C65—C66	0.6 (4)
N1—C6—C61—C62	130.5 (2)	C64—C65—C66—C61	−1.5 (4)
